# Prefrontal-Amygdala Connectivity and State Anxiety during Fear Extinction Recall in Adolescents

**DOI:** 10.3389/fnhum.2017.00587

**Published:** 2017-12-04

**Authors:** Despina E. Ganella, Marjolein E. A. Barendse, Jee H. Kim, Sarah Whittle

**Affiliations:** ^1^Behavioural Neuroscience Division, Florey Institute of Neuroscience and Mental Health, Parkville, VIC, Australia; ^2^Florey Department of Neuroscience and Mental Health, University of Melbourne, Parkville, VIC, Australia; ^3^Melbourne Neuropsychiatry Centre, Department of Psychiatry, University of Melbourne and Melbourne Health, Parkville, VIC, Australia

**Keywords:** adolescence, prefrontal cortex, amygdala, extinction, connectivity, anxiety

## Abstract

While deficits in fear extinction recall have been suggested to underlie vulnerability to anxiety disorders in adolescents, the neurobiology of these deficits remain underexplored. Here we investigate the functional connectivity (FC) of the ventromedial prefrontal cortex (vmPFC) and dorsolateral PFC (dlPFC) underlying extinction recall in healthy adolescents, and assess associations between FC and state/trait anxiety. Adolescents (17) and adults (14, for comparison) completed a fear-learning paradigm involving extinction and extinction recall during a functional magnetic resonance imaging session, in which skin conductance response (SCR) was recorded. Psychophysiological interaction analyses revealed that during extinction recall there was significant negative connectivity between the vmPFC and amygdala in adults, but not adolescents. vmPFC-amygdala connectivity was positively correlated with SCR. Adolescents showed significant negative FC between the dlPFC and the left and right hippocampus, and the amygdala, which was positively correlated with state anxiety. Recall was also associated with negative connectivity between the dlPFC and thalamus, posterior cingulate cortex, fusiform gyrus, and pallidum in adolescents. These results demonstrate that fear extinction recall in healthy adolescents is associated with FC between prefrontal and limbic brain regions, and suggest that alterations in connectivity may be associated with vulnerability to anxiety in adolescence.

## Introduction

Adolescence is a critical time for the development of anxiety disorders, with 75% being diagnosed during this period (Kessler et al., 2007). Adolescent onset of anxiety disorders leads to more severe impairment compared to adult onset (Newman et al., 1996). Despite these findings, adequate treatments for anxiety disorders are lacking, with over 50% of young people with anxiety disorders not responding to first-line treatments (Southam-Gerow et al., 2001). Adolescence represents a period of dramatic brain development and remodeling, which is suggested to contribute to an increased vulnerability to anxiety disorders (Cartwright-Hatton, 2006). However, there are substantial gaps in our knowledge about the biological mechanisms underlying anxiety disorders during adolescence, and it has been proposed that this is a key reason for the lack of progress in identifying effective treatments (Pine et al., 2009). Therefore, understanding how key factors underlying anxiety disorders are represented in the adolescent brain is essential for a better understanding of anxiety vulnerability and treatment.

One of the key underlying features of anxiety disorders and their treatment is the failure to appropriately inhibit, or extinguish, fear (Rothbaum and Davis, 2003; Otto et al., 2004; Pine et al., 2009). Fear extinction describes the decrease in a conditioned fear response (CR) after repeated exposure to a feared conditioned stimulus (CS), in the absence of the aversive unconditioned stimulus (US) with which it had been previously associated. Extinction recall is measured by presenting the CS alone after some interval of time following extinction. Low CR indicates effective extinction, while high CR indicates impaired extinction. Preclinical rodent studies have shown that although adolescents demonstrate successful within session extinction, they show failures in remembering extinction compared to older and younger rodents (McCallum et al., 2010; Kim et al., 2011; Pattwell et al., 2012; Zbukvic et al., 2017). Although less work has been done in humans adolescents, an extinction impairment has also been identified (Pattwell et al., 2012). Further, research has shown that fear extinction is associated with anxiety in young people (Jovanovic et al., 2014). Thus, extinction appears to be particularly impaired in adolescence, and it is possible that such deficits may represent a vulnerability to the development of anxiety during this time (see Kim and Ganella, 2015 for review).

Rodent studies have shown that adolescent deficits in extinction recall are due to altered plasticity in ventromedial prefrontal cortex (vmPFC) (Kim et al., 2011). For example, adolescent mice showed a lack of enhancement in glutamatergic synaptic transmission during extinction in the vmPFC compared to adult and preadolescent mice (Pattwell et al., 2012). These impairments may be partly due to the dramatic maturation that occurs in the adolescent brain, particularly in relation to connectivity in neural networks involving the prefrontal cortex (Casey et al., 2005). Indeed, extinction learning and recall depend on the integrated functioning of a neural circuit involving the amygdala, vmPFC, and the hippocampus (see Corcoran and Quirk, 2007 for review). The amygdala is important for emotional reactivity and the storage of fear memories, while the vmPFC appears critical for the consolidation and expression of extinction memory (Quirk et al., 2000; Sotres-Bayon et al., 2006), and the suppression of fear after extinction (Milad and Quirk, 2002; Quirk et al., 2006; Zbukvic et al., 2016). This is thought to occur via connections from the vmPFC to inhibitory neurons in the amygdala which when activated inhibit fear expression (Herry et al., 2008). The hippocampus is commonly associated with processing contextual information to modulate fear expression within the extinction context (Corcoran et al., 2005), and it has been suggested that the hippocampus controls the context-specific retrieval of extinction through projections to the vmPFC (Corcoran and Quirk, 2007). Finally, the dorsolateral (dl) PFC has recently been identified as having an important role in fear regulation, through cognitive emotion regulation strategies (see Hartley and Phelps, 2010 for review).

Although limited, there is some evidence that in healthy human *adults*, recall of extinguished fear activates the PFC, amygdala, and hippocampus in concert, and that activity in the vmPFC is positively associated with both amygdala and hippocampus activation during extinction recall (Milad et al., 2007). While there are a few MRI studies that have investigated aversive learning and responses to learned threat in adolescents (Britton et al., 2013; Tzschoppe et al., 2014), no human studies have investigated how extinction recall is associated with (FC) between these brain regions in adolescents, or how such connectivity might be associated with anxiety vulnerability.

Thus, the aim of this study was to investigate FC of the vmPFC and dlPFC with the amygdala and hippocampus during short-term fear extinction recall in healthy adolescents, and to assess whether such connectivity was associated with state and trait anxiety. An adult sample was also included for comparison purposes. We hypothesized that during recall, adolescents would show FC between the PFC (vmPFC and dlPFC) and amygdala and hippocampus. Although no other studies have investigated associations between anxiety and PFC in the context of extinction recall, given evidence from other research that elevated anxiety is associated with increased connectivity between the PFC and amygdala and hippocampus during aversive learning (Tzschoppe et al., 2014), we hypothesized that PFC connectivity with the amygdala and hippocampus during recall would be increased in those with higher levels of anxiety.

## Methods and materials

### Participants

All participants (and their parents if <18 years of age) provided written informed consent to participate in the study, which was approved by the Royal Children's Hospital Research Ethics Committee: 34141A, Victoria, Australia. Eighteen healthy adult participants (aged 25–35) were recruited from the community and 20 healthy adolescents (aged 14–16) were recruited from schools in Melbourne, Australia. Exclusion criteria included (i) current treatment for a psychiatric illness, (ii) non-native English speaker, (iii) current psychoactive medication use, (iv) pregnant, and (v) contraindications to MRI. Data from 14 adults (6 females, M age 29.85 years, S.D. 3.03 years) and 17 adolescents (10 females, M age 16.26 years, S.D. 0.4 years) were included in analyses after exclusions based on technical scanner issues (*n* = 1 adult), image acquisition problems (*n* = 1 adult) and excessive head motion (*n* = 2 adults, *n* = 3 adolescents).

### Magnetic resonance imaging assessment: image acquisition

Neuroimaging data were acquired on a 3T Siemens TIM Trio scanner (Siemens, Erlangen, Germany) at the Murdoch Children's Research Institute, Royal Children's Hospital, Melbourne, Australia. Participants lay supine with their head supported in a 32-channel head coil and headphones. For stage one of the task (Conditioning and Extinction), 296 whole-brain T2^*^-weighted echo-planar images [(repetition time (TR) = 3,000 ms, echo time (TE) = 40 ms, pulse angle = 85°, field of view (FOV) = 216 mm] were acquired, corresponding to 40 interleaved slices with a voxel size of 3 × 3 × 3 mm. For stage two of the task (Recall/Reconditioning and Re-extinction) 170 whole-brain T2^*^-weighted echo-planar images with the same parameters as stage one were acquired. T1-weighted MPRAGE images were also acquired for co-registration purposes (TR = 2,530 ms, TE = 1.74 ms, flip angle = 7°, FOV = 256 × 208 mm), producing 176 1 mm contiguous sagittal slices [voxel dimensions = 1 mm (Southam-Gerow et al., 2001)]. Note that the analyses of conditioning, extinction, and re-extinction phases are the subject of a separate publication. The focus of the present study is on FC during extinction recall, specifically.

### Event related paradigm design

The task was presented with Paradigm software (http://www.paradigmexperiments.com), running on a Dell computer. The LCD screen that presented stimuli was visible via a reverse mirror mounted to the participants' head coil and skin conductance response (SCR)s were acquired throughout.

The fear conditioning paradigm we used was based on that by Britton et al. (2013). The paradigm was run in the scanner over 45 min and involved two consecutive stages, each with two phases. Stage 1: Conditioning, where one of two neutral faces from the NIMSTIM (Tottenham et al., 2009) set (CS+) was presented for 3 s followed by 2–4 s jittered trace period then a fear face (1 s) and female scream (US, ~95–100 dB) on 100% of trials. This has been shown to lead to robust subjective rating of anxiety to the CS in adolescents and adults (Britton et al., 2011, 2013; Lau et al., 2011). The jittered trace interval was chosen based on Lau et al. (2011) and Britton et al. (2013) for optimal fMRI analyses and to reduce US habituation (Lake et al., 2016). The CS+ was reinforced with the US 100% during conditioning based on other human fear trace conditioning studies (for review see Sehlmeyer et al., 2009; Fullana et al., 2016). Given the trace period, responses to the CS+ were not confounded with the US, and thus 100% reinforcement was not problematic. The other face (CS–, 3 s) was used as a control stimulus that was never paired with the US. CS+US trials and CS– trials were interleaved and were presented 15 times each in random order. The inter-trial interval (ITI) was a white fixation cross on a black background, jittered for 8–12 s. Extinction followed immediately, 15 CS+ (3 s) trials in the absence of the US, randomly interleaved with 15 CS– (3 s) trials. After extinction there was a ~10 min rest prior to recall (based on similar timing used in previous studies; LaBar and Phelps, 2005). Stage 2: Recall and Re-conditioning trials were given, where the CS+US and CS– (one of each for Recall, one of each for Re-conditioning) were randomly presented, followed by a Re-extinction phase, which was identical to the extinction phase. The two faces were counterbalanced as CS+ or CS– and there were no more than two consecutive trials of the CS+ or CS–. Familiarization of the task was conducted outside the scanner, where participants were presented with two neutral female faces from the NIMSTIM set (Tottenham et al., 2009), which were different to the faces presented in the scanner.

### Measures of skin conductance response and anxiety levels

SCR was collected throughout the fMRI paradigm using a galvanic skin response amplifier (ADInstruments, Milford, MA), an ADInstruments Powerlab acquisition system, and extracted and analyzed using ADInstruments labchart software. Two electrodes were attached with a Velcro strap (and electrolyte gel) to the palmar surfaces of the index and middle fingers of the non-dominant hand. SCRs to each CS+ and CS– were identified by the peak skin conductance level in the 5 s from CS onset (Pattwell et al., 2012). Across all the phases, the peak SCR value for each CS+ or CS– was never confounded with the US trial that always began 5+ s from CS onset. Analyses were then carried out on difference scores in SCR (CS+ minus CS– Pattwell et al., 2012). Note that we only report and discuss results for Extinction Recall here, given the aims of the study. It is also important to note that we found no significant age-related differences in SCR during conditioning, extinction or re-extinction (Ganella et al., under review), please see Supplementary Table 1 for average SCR for each phase of the paradigm. In addition, immediately prior to the MRI, participants completed a short battery of questionnaires, including the Spielberg State-trait Anxiety Inventory (STAI) (Spielberger et al., 1983), which was used to assess state and trait anxiety.

### fMRI statistical analyses

Four “dummy” volumes acquired at the beginning of each session were discarded to allow for T1 equilibration effects. We performed pre-processing procedures using Statistical Parametric Mapping (SPM) 12 (http://www.fil.ion.ucl.ac.uk/spm/). These included slice timing correction, motion correction, co-registration of functional images with participants' T1-weighted image, which had been co-registered to the SPM-T1 template. Co-registered volumes were concurrently re-sliced to 2 mm isotropic resolution and normalized to SPM-T1 template. The resulting transformation matrix was applied to the functional data to achieve accurate spatial normalization across individuals. Finally, functional images were smoothed using a Gaussian filter (full-width at half maximum, 6 mm). Head motion was inspected for each participant; maximal amplitude of translational and rotational displacements (x, y, z) were required to be <3 mm or 3°, respectively, for all participants, and those which exceeded this threshold were excluded from analyses as reported in other studies (Ginther et al., 2016; Cignetti et al., 2017).

### Analysis of functional connectivity

To investigate FC we selected the right hemisphere vmPFC and dlPFC as seed regions of interest (ROIs). We used psychophysiological interaction (PPI) analysis (Friston et al., 1997) to estimate connectivity between vmPFC and dlPFC and our target ROI (amygdala and hippocampus) during extinction recall. We also conducted an exploratory whole brain connectivity analysis to identify regions of significant FC outside of the amygdala and hippocampus. Initially, first-level whole-brain analyses were conducted to create contrast images comparing the CS+ to the CS– during extinction recall. Motion parameters were included as covariates. A high-pass filter set at 128 s was applied to remove low-frequency drifts. Second, for each individual, the average time series of neural activation was extracted from each ROI and entered as a physiological variable in the PPI model. ROIs (vmPFC and dlPFC) were 6 mm spheres around MNI coordinates of brain regions that showed peak neural activation that was lower in adolescents compared to adults for the contrast, recall > late extinction, in a previous study from the same sample (Ganella et al 2017, under review). The task contrast of interest [recall (CS+ > CS–)] was entered as a psychological variable. The psycho-physiological interaction was obtained by modeling a third variable as the interaction term between the latter two variables. PPI-interaction terms were created for all individuals and fed into first-level whole-brain, linear regression analyses. This resulted in contrast images for all participants showing the effect of the PPI-interaction.

### Second-level analyses

Following processing at the first-level, the above mentioned contrast images were included in second-level random effects analyses to assess within age group effects. Second-level results were corrected for multiple analyses using a cluster forming threshold of *p* < 0.005 and a cluster-level threshold of *p* < 0.05 family-wise error (FWE) correction as determined by AFNI's 3dClustSim program (version 16.3.09) using 20,000 iterations, a mask of the whole brain, and a smoothness estimated using 3dFWHMx with the–ACF option. The minimum threshold for whole brain analyses was 337 voxels. Given priori hypotheses about the role of the amygdala and hippocampus, bilateral masks of these regions were created using the WFUpickatlas toolbox (http://www.fmri.wfubmc.edu/cms/software) (Maldjian et al., 2003). Clusters within these ROI were thresholded to achieve a small volume corrected *p-*value of 0.05, as determined by 3dClustSim (using the same parameters as described above, including a cluster forming threshold of *p* < 0.005). The minimum threshold for the amygdala and hippocampus were 10 and 18 voxels, respectively.

From each individual in both groups, betas were extracted for any significant clusters resulting from the within age group connectivity analyses. Betas from spheres with a radius of 4 or 6 mm (depending on region size) centered on the peak coordinates of significant clusters were extracted for plotting (for visualization purposes) and performing Pearson bivariate correlations (GraphPad Prism 6) to investigate whether there were any relationships between FC and state and trait anxiety scores and SCR. Because age group differences were not a primary aim, between-age group effects were not assessed at the voxel-wise level. Please note, for figures that show bar graphs of the significant clusters, bar graphs are only for illustration, and not inferential purposes.

## Results

See Table 1 for descriptive information about the sample. Trait (but not state) anxiety scores and SCR during recall were significantly higher in adolescents compared to adults (trait anxiety, *p* = 0.023; SCR, *p* = 0.0016).

**Table 1:** Descriptive sample information.

	**Number**	**Age (years) (mean, S.D.)**	**Gender (males, females)**	**State anxiety score (mean, S.D.)**	**Trait anxiety score (mean, S.D.)**	**Recall skin conductance response (CS+ - CS−)**
Adults	14	29.9, 3.0	(8, 6)	46.2, 6.1	45.6, 3.4	−0.648, 0.572
Adolescents	17	16.3, 0.4	(7, 10)	47.1, 4.9	48.3, 3.4	0.068, 0.542

*Mean state anxiety score for adults after windsorizing 1 statistical outlier (>3 S.D. below the mean) = 47.0, S.D. = 3.8*.

### vmPFC seed region

**Figure 1:**
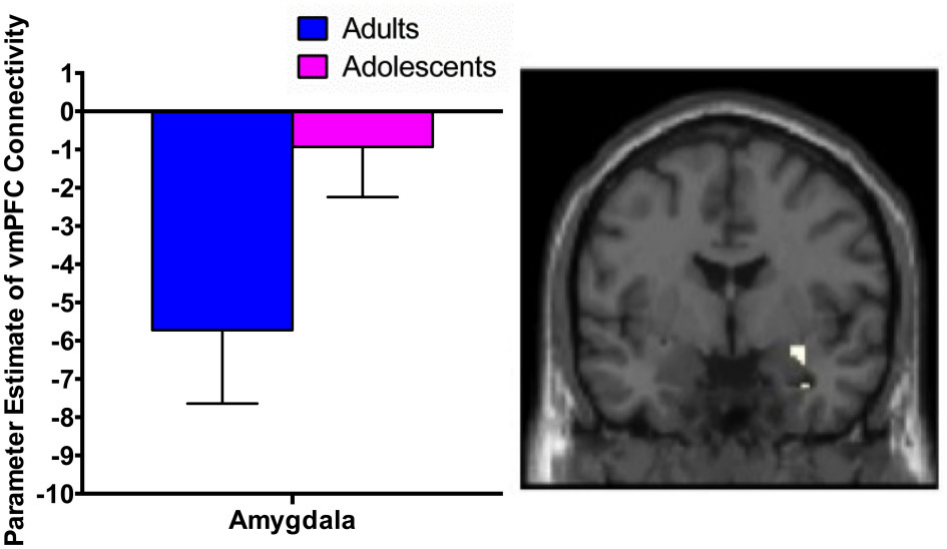
vmPFC functional connectivity with the amygdala during extinction recall. Adults showed significant negative functional connectivity with the right amygdala. Bar graphs show extracted parameter estimates of right vmPFC-amygdala connectivity generated from a 4 mm sphere around the peak coordinates of the significant amygdala cluster.

While extinction recall was not associated with vmPFC connectivity in adolescents, there was significant negative connectivity between the right vmPFC and right amygdala in adults (Table 2, Figure 1).

**Table 2:** Significant connectivity results for vmPFC and dlPFC seed regions.

	**Hemisphere**	**Voxels (n)**	**t**	**x**	**y**	**z**
**vmPFC seed region**	R			14	48	−6
Adults (negative connectivity)	R	24	3.20	30	−2	−14
Amygdala						
**dlPFC seed region**	R			36	44	10
Adolescents (negative connectivity)	R	27	3.48	34	2	−20
Amygdala						
Hippocampus	L	23	3.90	−24	−18	−14
Hippocampus	R	18	3.46	32	−36	−6
Thalamus	R	388	4.80	4	−8	6
Fusiform gyrus	L	339	4.76	−40	−50	−16
Pallidum	R	360	4.66	24	−2	−6
Occipital fusiform gyrus	R	1447	3.88	34	−64	−14
Posterior cingulate cortex	L	702	4.64	−2	−52	18

*dlPFC, dorsolateral prefrontal cortex; vmPFC, ventromedial prefrontal cortex; L, left hemisphere; R, right hemisphere. Note that there were no significant positively valenced connectivity results. x, y, z represent MNI coordinates*.

*All results survived cluster correction p < 0.05 using a cluster-forming threshold of p < 0.005. Results for both masked (amygdala and hippocampus) and whole brain analyses are presented*.

### dlPFC seed region

**Figure 2:**
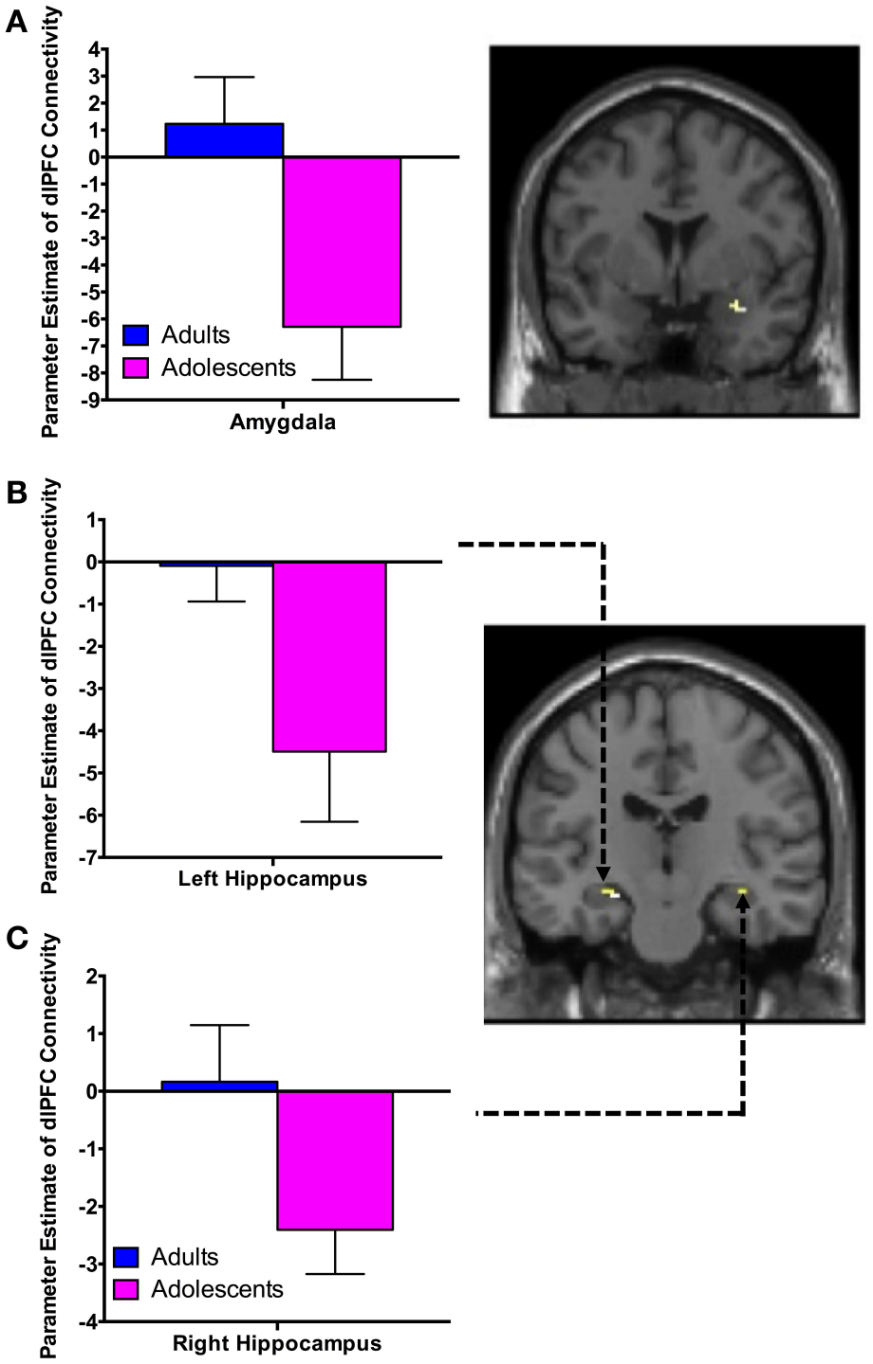
dlPFC functional connectivity with the amygdala and hippocampus during extinction recall. Adolescents showed significant negative connectivity between the right dlPFC and right amygdala **(A)**, the right dlPFC and left hippocampus **(B)** and the right dlPFC and right hippocampus **(C)**. Bar graphs show extracted parameter estimates of dlPFC-amygdala and dlPFC-hippocampus connectivity generated from a 4 mm sphere around the peak coordinates of the significant clusters. Note that a statistical outlier (>3 S.D. below the mean) for dlPFC-hippocampus connectivity (left and right) was windsorized prior to plotting.

**Figure 3:**
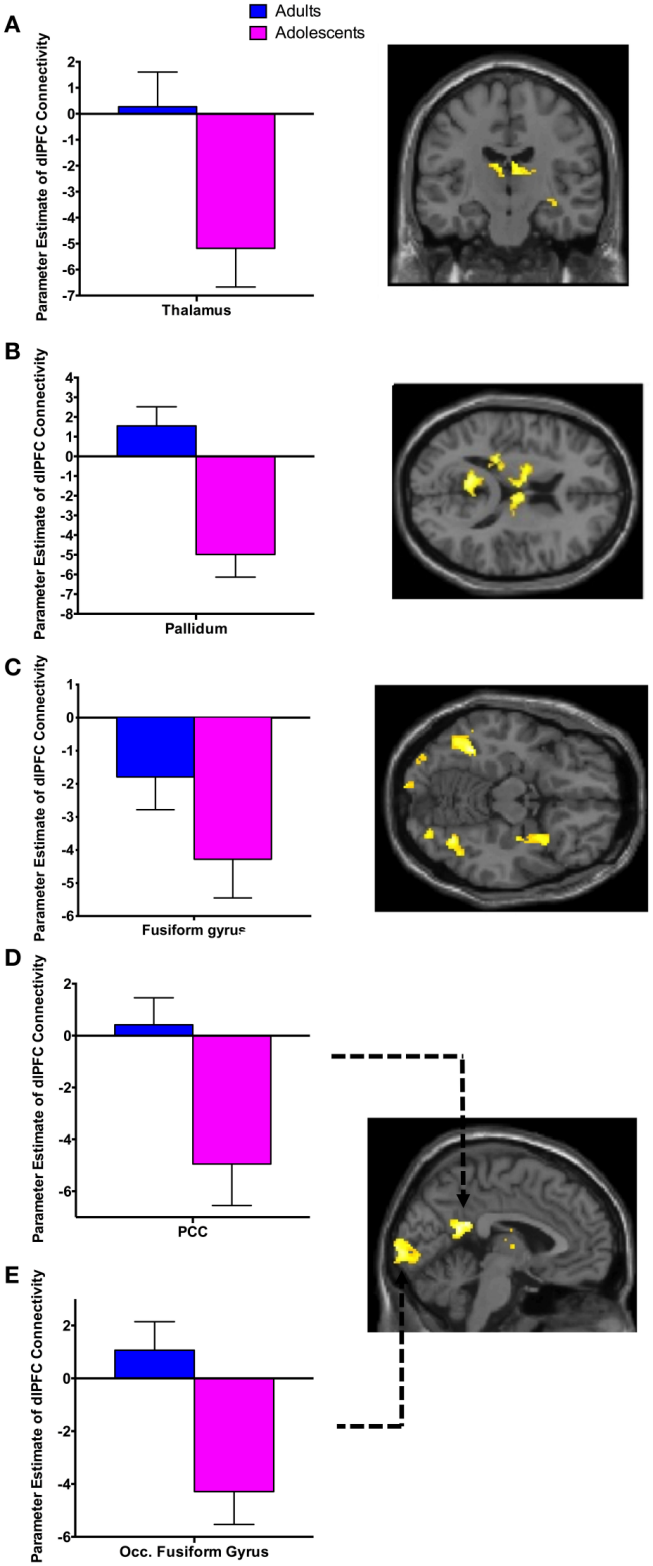
Whole brain analysis of functional connectivity with the dlPFC seed during extinction recall. Whole brain exploratory analysis revealed significant negative connectivity in adolescents between the right dlPFC and the thalamus **(A)**, pallidum **(B)**, fusiform gyrus **(C)**, posterior cingulate cortex (PCC) **(D)** and occipital fusiform gyrus **(E)**. Bar graphs show extracted parameter estimates of dlPFC connectivity with their respective regions, generated from a 4 mm sphere (thalamus and pallidum) or 6 mm sphere (PCC, occipital fusiform gyrus and fusiform) around the peak coordinates of the significant clusters. Note that a statistical outlier (>3 S.D. below the mean) for dlPFC connectivity with the PCC, occipital fusiform gyrus, and fusiform gyrus was windsorized prior to plotting.

There was significant negative connectivity in adolescents between the right dlPFC and right amygdala (Table 2, Figure 2A). Adolescents also showed significant negative connectivity between the dlPFC and hippocampus in both hemispheres (Table 2, Figures 2B,C). In our whole brain analysis we found significant negative connectivity in adolescents between the dlPFC and PCC, occipital fusiform gyrus, thalamus, pallidum and fusiform gyrus (Table 2, Figure 3).

### Correlation analyses

**Figure 4:**
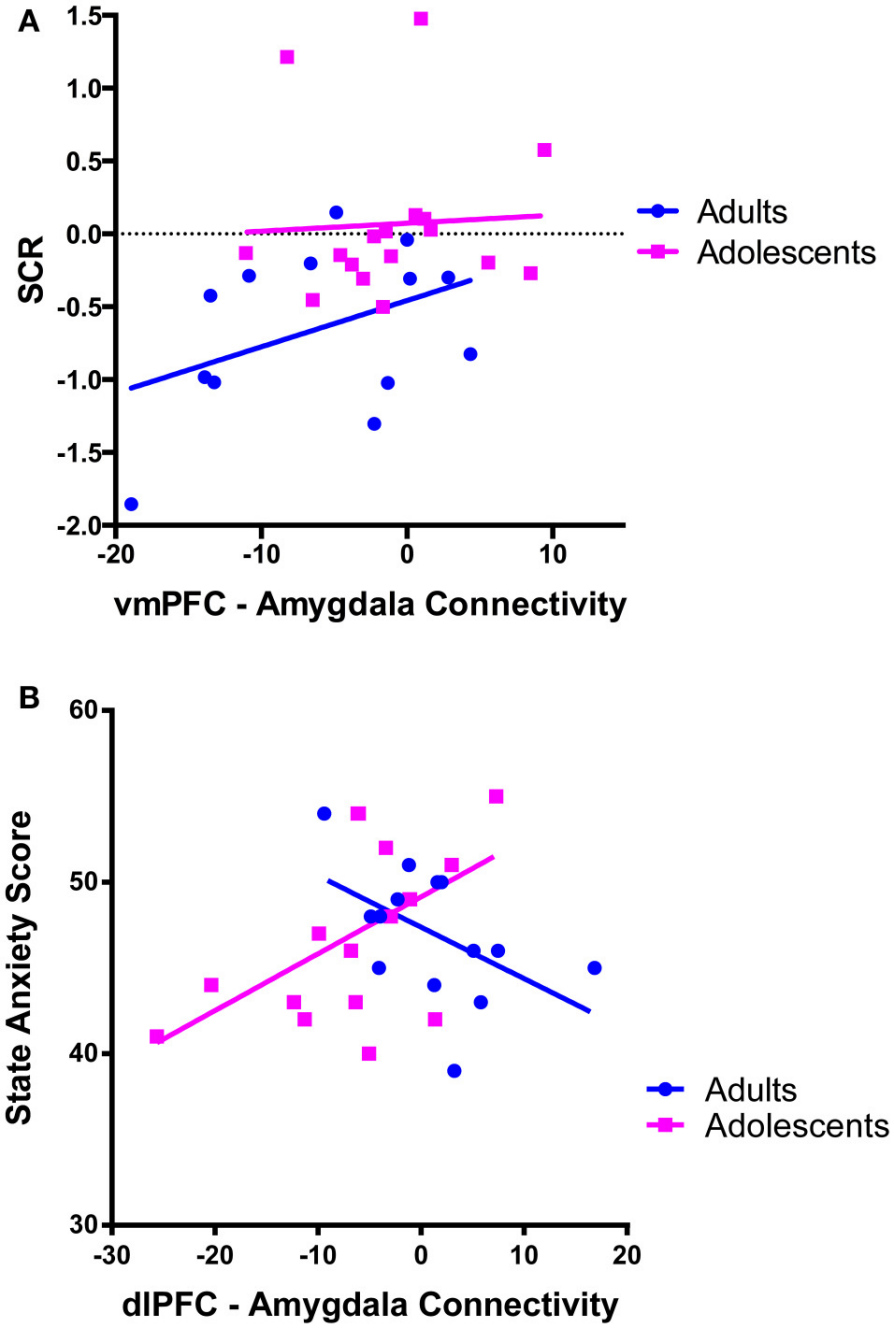
Correlations between prefrontal-amygdala connectivity with anxiety and skin conductance response. **(A)** There was a trend for vmPFC-amygdala connectivity to correlate with skin conductance response (SCR) during extinction recall [SCR was calculated as the difference score (CS+ minus CS–)] in adults *r* = 0.412, *p* = 0.162, but not adolescents *r* = 0.055, *p* = 0.834. **(B)** dlPFC-amygdala connectivity correlated with state anxiety symptoms in adolescent participants. There was a positive association between dlPFC-amygdala connectivity and state anxiety in adolescents (*r* = 0.545, *p* = 0.024). The association in adults was not significant when a statistical outlier (>3 S.D. below the mean) was included (*r* = −0.364, *p* = 0.201) or windsorized (*r* = −0.511, *p* = 0.062).

There was a trend for a significant positive correlation between SCR during recall (discrimination score between CS+ and CS–) and vmPFC-amygdala connectivity in adults (Figure 4A).

We observed a significant positive correlation between state anxiety and dlPFC-amygdala connectivity in adolescents (*p* = 0.024; Figure 4B).

## Discussion

In the present study, extinction recall was not associated with vmPFC connectivity in adolescents. However in adults, we observed significant negative (i.e., inverse) vmPFC-amygdala connectivity, which was correlated with SCR. In addition, we found significant negative connectivity between the dlPFC and the amygdala and hippocampus during recall of extinguished fear in adolescents. dlPFC-amygdala connectivity was correlated with state anxiety in adolescents such that stronger inverse connectivity was associated with reduced symptoms. Whole brain exploratory analyses also revealed significant negative connectivity between the dlPFC and PCC, occipital fusiform gyrus, fusiform gyrus, pallidum, and thalamus, in adolescents. Together, these findings illustrate that prefrontal brain connectivity may underlie deficits in extinction recall in adolescents, which could contribute to anxiety vulnerability in this age group.

We observed significant negative connectivity between the vmPFC and amygdala in adults. Milad et al. also identified significant coupling between the vmPFC and amygdala during extinction recall in adults, however they found a positive functional correlation (Milad et al., 2007). They proposed that this was reflective of activation of inhibitory circuits within the amygdala in conjunction with vmPFC activation to successfully reduce fear expression (Milad et al., 2007). Although we cannot determine directionality from our PPI analyses, the negative connectivity between the vmPFC and amygdala that we observed may be consistent with other literature that posits that the PFC has a top-down inhibitory effect on amygdala reactivity (Hariri et al., 2003; Kim et al., 2003; Hare et al., 2008), based on findings of an inverse correlation between vmPFC activity and amygdala activation upon presentation of emotional stimuli and regulation of negative affect (Kim et al., 2003; Shin et al., 2004; Urry et al., 2006). The amygdala-mPFC circuit is a primary neural substrate of emotion processing and regulation, and is disrupted in anxiety disorders (Phelps et al., 2001; Hariri et al., 2003; Shin et al., 2004; Phan et al., 2005; Hare et al., 2008), however, the relationship between these two brain regions during recall of extinguished fear is not well understood. Negative FC in adults may be an adaptive mechanism associated with increased ability to recall that a fear-inducing stimulus is extinguished (e.g., greater top-down control of amygdala reactivity by the vmPFC). This is supported by our observation that stronger negative connectivity was significantly associated with lower SCR during recall, which is indicative of reduced fear to the CS+ at the behavioral level. This process may be less efficient during adolescence, which is consistent with reports that there is ongoing development and maturation of vmPFC-amygdala resting state connectivity (Gabard-Durnam et al., 2014). Although we found no links between adolescent SCR (or state or trait anxiety) and vmPFC-amygdala FC, the absence of connectivity in these brain regions in adolescents may underlie the general impairment in fear extinction recall and the increased risk for anxiety disorders during this period (Merikangas et al., 2010).

We observed significant negative FC between the dlPFC and amygdala in adolescents. Although dlPFC activity or connectivity with the amygdala has not traditionally been emphasized as critical for extinction recall, it has recently been identified as having an important role in fear regulation, through cognitive emotion regulation strategies (Hartley and Phelps, 2010). While this dlPFC-amygdala connectivity was not associated with SCR, it was positively correlated with state anxiety in adolescents, whereby stronger negative connectivity was associated with less state anxiety (or in other words, less negative connectivity is associated with higher state anxiety). This finding is consistent with hypotheses, and with other research by Tzschoppe et al. showing a positive correlation between dlPFC-amygdala connectivity and neuroticism in adolescent participants during aversive learning (Tzschoppe et al., 2014). It is of note that Tzschoppe et al. also reported that state anxiety and vmPFC-amygdala connectivity was positively correlated in adolescents, which we did not observe (Tzschoppe et al., 2014). This inconsistency may be due to differences in sample size, or the differing nature of the tasks.

It is possible that the significant negative dlPFC connectivity observed in our adolescent participants (at the group level) may be adaptive at this particular age. dlPFC-amygdala connectivity in adolescents may reflect a compensatory mechanism to regulate amygdala reactivity compared to vmPFC-amygdala connectivity in adults (Hariri et al., 2003; Kim et al., 2003; Hare et al., 2008). However, if stronger dlPFC-amygdala connectivity is adaptive in adolescents, it is unclear why adolescents in general exhibit extinction recall deficits (as per previous research McCallum et al., 2010; Kim et al., 2011 and our present SCR findings). Given the consistently reported role of the dlPFC in engagement of active self-regulation of negative emotional stimuli, which is in turn associated with attenuation of limbic-amygdala responses (Hariri et al., 2000; Taylor et al., 2003; Etkin et al., 2006; Banks et al., 2007), the connectivity profile we observed may be reflective of an ineffective attempt at top-down control necessary for extinction recall in adolescents. However, this is speculative and requires further investigation.

In adolescents, there was significant negative connectivity between the dlPFC and hippocampus in both hemispheres during extinction recall. The hippocampus is an important component of the corticolimbic circuitry involved in learning and memory (Eichenbaum, 1997), and it undergoes robust maturation well into adolescence (Giedd et al., 1996; Suzuki et al., 2005). While it has a well-established role in retrieving contextual information on the extinction memory (Milad et al., 2007; Orsini et al., 2011), given that we did not alter the recall context in our fear-conditioning paradigm, our finding may be more reflective of the general role of the hippocampus in declarative or episodic memory processes (Tulving and Markowitsch, 1998). Significant negative dlPFC-hippocampus connectivity may be reflective of attempts to suppress negative memories in adolescents (Benoit et al., 2014). This is consistent with work by Benoit and colleagues, who found that negative dlPFC-hippocampus connectivity was associated with suppression and better coping with intrusive memories (Benoit et al., 2014).

Differences observed between adolescents and adults in the current study may be due to functional and structural maturation of the brain during adolescence. During adolescence there is a dramatic period of cortical synaptic pruning, with the PFC being one of the last brain regions to mature (Lenroot and Giedd, 2006; Casey et al., 2008). In contrast the amygdala and hippocampus follow a more linear (positive) developmental trajectory. It has been suggested that this differential development creates a mismatch whereby subcortical regions such as the amygdala mature early, while the PFC continues to develop (Casey et al., 2008; Shaw et al., 2008). Therefore, a relative immaturity of the PFC may result in less effective top-down regulation, which may lead to impaired emotion processing and regulation of fear memories during adolescence. Further, some research suggests that ventral vs. dorsal PFC structure develops at different rates in adolescence (Markham et al., 2007), which may explain dissociated results between the vmPFC vs. dlPFC in our study. More research is required to understand the relevance of differential maturation of these regions for extinction recall and anxiety in adolescents vs. adults.

Connectivity between the dlPFC and a number of brain regions other than our hypothesized ROIs during recall in adolescents but not adults was unexpected. Adolescents displayed significant negative FC between the dlPFC and the PCC, fusiform gyrus, thalamus, pallidum, and occipital fusiform gyrus. This may also be a consequence of the protracted development of the PFC during this age. Experience-dependent synaptic pruning of the PFC is believed to be essential for the fine-tuning of functional neural networks during adolescence, rendering the remaining synaptic circuits more efficient (Blakemore and Choudhury, 2006). It may be the case that less refinement in dlPFC connectivity results in more engagement of brain regions during recall. This may be indicative of a compensatory strategy used while the brain is less efficient in integrating executive functions.

One limitation of the present study is the relatively small number of participants that were included. Given lack of current tools, we were unable to conduct a power analysis to inform an appropriate sample size for our connectivity analyses. However, the effect sizes of the associations between vmPFC/hippocampal activity and behavioral response during extinction recall found by Milad et al. suggests that we may have had adequate power (i.e., >80%) to examine correlations between connectivity metrics and SCR/anxiety. Of note is that effect sizes from other relevant research (e.g., correlations between trait anxiety and amygdala-PFC connectivity during fear face processing Robinson et al., 2012) suggest lower power estimates. As such, future research with larger sample sizes is likely needed to replicate our findings. A second limitation is that gonadal hormones are likely influencing emotion regulation during this stage of pubertal development, which may impact fear learning and extinction. It would be informative to expand our study in order to elucidate gender differences and the role of sex hormones on connectivity between brain regions during fear extinction recall in adolescents. Finally, PPI analysis allows us to observe correlated brain function, but cannot shed light on directionality of influence of one brain region on another.

We have demonstrated that fear extinction recall in adolescents is associated with FC between prefrontal brain regions and limbic and cortical brain regions, and we speculate that such connectivity may collectively contribute to fear extinction recall deficits observed in adolescents. Further, links between dlPFC-amygdala connectivity and state anxiety in adolescents contribute to a better understanding of the neural circuitry underlying risk for anxiety disorders. Findings may reflect different rate of maturation of emotional compared to cognitive systems, which would have consequences for mental health (Steinberg, 2005). Our findings call for a better understanding of the development of the prefrontal cortex during this developmental stage in order to tailor effective treatments to adolescents with anxiety.

## Author contributions

DG, JK, and SW designed the study, analyzed and interpreted the data and wrote the manuscript. DG collected all the data. MB assisted in the analysis of data and drafting of the manuscript.

### Conflict of interest statement

The authors declare that the research was conducted in the absence of any commercial or financial relationships that could be construed as a potential conflict of interest.
