# Supplementary material for: Prefrontal-Amygdala Connectivity and State Anxiety during Fear Extinction Recall in Adolescents
Source: Front Hum Neurosci. 2017 Dec 4;11:587. doi: 10.3389/fnhum.2017.00587 (PMC5722839; doi:10.3389/fnhum.2017.00587)
Supplement: Supplementary file 1 [file Table1.docx]

**Supplementary Table 1. Skin Conductance Response data**

|  | Early Conditioning | Late Conditioning | Early Extinction | Late Extinction | Recall | Re-conditioning | Early Re-extinction | Late Re-extinction |
| --- | --- | --- | --- | --- | --- | --- | --- | --- |
| Adults | -0.059,  0.138 | 0.173,  0.117 | -0.003,  0.104 | -0.030,  0.224 | -0.648, 0.572 | -0.049,  0.239 | 0.035,  0.075 | 0.055,  0.110 |
| Adolescents | 0.002,  0.172 | 0.103,  0.147 | 0.053,  0.166 | -0.072,  0.250 | 0.068, 0.542 | -0.299,  0.487 | -0.033,  0.138 | -0.038,  0.177 |

Note: Across each phase, the peak SCR value for each CS+ or CS- was measured, these peak values were then averaged across blocks of 5 CS+ and 5 CS- for each phase of the paradigm, except for recall and re-conditioning phase for which there was only a single CS+ and single CS-. Data represents difference scores (CS+ minus CS-) at various phases of the paradigm (Mean, S.D.).
